# Protective Role of Low Ethanol Administration Following Ischemic Stroke via Recovery of KCC2 and p75^NTR^ Expression

**DOI:** 10.1007/s12035-020-02176-x

**Published:** 2020-10-24

**Authors:** Stanislav Khirug, Shetal Soni, Marta Saez Garcia, Marine Tessier, Liang Zhou, Natalia Kulesskaya, Heikki Rauvala, Dan Lindholm, Anastasia Ludwig, Florence Molinari, Claudio Rivera

**Affiliations:** 1 Neuroscience Center-HiLIFE, University of Helsinki, 00014 Helsinki, Finland; 2 INMED (INSERM U1249), Aix-Marseille Université, Marseille, France; 3 Medicum, Department of Biochemistry and Developmental Biology, Faculty of Medicine, University of Helsinki, Helsinki, Finland; 4 Minerva Foundation Institute for Medical Research, Biomedicum Helsinki 2U, Helsinki, Finland

**Keywords:** Apoptosis, Trauma, Chloride homeostasis, GABA_A_ transmission, Neurotrophines

## Abstract

A striking result from epidemiological studies show a correlation between low alcohol intake and lower incidence for ischemic stroke and severity of derived brain injury. Although reduced apoptosis and inflammation has been suggested to be involved, little is known about the mechanism mediating this effect in vivo. Increase in intracellular chloride concentration and derived depolarizing GABA_A_R-mediated transmission are common consequences following various brain injuries and are caused by the abnormal expression levels of the chloride cotransporters NKCC1 and KCC2. Downstream pro-apoptotic signaling through p75^NTR^ may link GABA_A_ depolarization with post-injury neuronal apoptosis. Here, we show that changes in GABAergic signaling, Cl^−^ homeostasis, and expression of chloride cotransporters in the post-traumatic mouse brain can be significantly reduced by administration of 3% ethanol to the drinking water. Ethanol-induced upregulation of KCC2 has a positive impact on neuronal survival, preserving a large part of the cortical peri-infarct zone, as well as preventing the massive post-ischemic upregulation of the pro-apoptotic protein p75^NTR^. Importantly, intracortical multisite in vivo recordings showed that ethanol treatment could significantly ameliorate stroke-induced reduction in cortical activity. This surprising finding discloses a pathway triggered by low concentration of ethanol as a novel therapeutically relevant target.

## Introduction

The increasing global prevalence of stroke is a serious concern for society, with 10.3 million strokes annually (67% ischemic strokes, IS). In addition to high mortality rates, stroke-induced debility accounts for a large share of societal burden: in 2013, stroke was responsible for 113 million disability-adjusted life years (DALYs) worldwide [1]. At present, only one FDA-approved therapy is available for acute ischemic stroke (AIS); IV recombinant tissue plasminogen activator (rtPA) therapy and its use are limited to just 5–10% of AIS patients [2]. All new avenues of treatment must subsequently be examined to allow additional options for AIS therapy.

A number of epidemiological studies have investigated the dose-dependency of alcohol intake, the risk, and severity of ischemic stroke [3–8]. Strikingly, these studies found a strong correlation between low intake and lower risk and severity of post-ischemic injury. Both effects in apoptosis and inflammation are proposed to be involved in the mechanism in rodents [9, 10].

Numerous neurological conditions, including temporal lobe epilepsy, traumatic brain injury, and spinal cord injury, have reported a depletion of the potassium-chloride cotransporter KCC2, in neurons proximal to the affected site [11–14]. KCC2 protein-mediated chloride extrusion is crucial for maintaining classical GABAergic hyperpolarizing inhibition in adult mature neurons [15]. The downregulation of KCC2 is therefore critical for the depolarizing effect of GABA in injured neurons [14, 16]. In a recent study on mice, application of just 3% ethanol to the drinking water for 5 days led to a significant increase in KCC2 levels but not NKCC1 [17].

This present work confirms that KCC2 protein levels in the plasma membrane of neurons are substantially reduced in the acute, focal ischemic mice model, as observed previously in other stroke models [18–21]. We subsequently accomplished a rescue of the KCC2 levels and GABA-mediated responses in neurons of the peri-infarct zone by supplementation of the drinking water with 3% ethanol for 5 days post-ischemia. Recent results show that pan-neurotrophin receptor p75 (p75^NTR^) activation can induce a downregulation of KCC2 in vivo [22] in addition to its well-established mediation of apoptotic signaling [23]. Intriguingly, here, we found that ethanol treatment significantly diminishes post-ischemic rise in p75^NTR^ levels and reduces neuronal death. The ethanol-dependent inhibition of post-ischemic p75^NTR^ upregulation correlates with KCC2 downregulation and qualitative changes in GABA_A_ responses, as well as neuronal death in the post-ischemic peri-infarct zone. This is followed by significant rescue of post-ischemic decrease in network activity in the core region.

## Materials and Methods

The University of Helsinki guidelines for experimentation on mice were carefully followed. The details of the experimental procedures were approved by the national ethics committee for Animal Research (ESAVI/18276/2018).

### Photothrombotic Model of Ischemic Stroke

Two-month-old C57BL6 male mice were used for this study. Mice were allowed free access to room air and were anesthetized with intraperitoneal injection of mixture of ketamine (80 mg/kg body weight) and xylazine (10 mg/kg) during surgery and stroke induction. The depth of anesthesia, blood oxygen saturation level (> 90%), and heart rate (450–650 beats/min) were monitored continuously with a MouseOx pulse oximeter (STARR Life Sciences) equipped with a mouse thigh sensor. A heating pad was used to maintain the core temperature of 37 °C. Implantation of cranial window was performed as described previously [24, 25]. Briefly, a subcutaneous injection of 0.1% lidocaine was administered to reduce local pain at the incision site, and then skin and connective tissue attached to the skull were removed. To form a round cranial window of ∼ 3 mm diameter, cranial bone over the somatosensory cortex was carefully removed. The brain was then covered with sterile cortex buffer containing the following (in mM): 125 NaCl, 5 KCl, 10 glucose, 10 HEPES, 2 CaCl_2_, and 2 MgSO_4_, pH 7.3, and a 5-mm diameter #1.5 glass coverslip (Electron Microscopy Sciences) was placed over the window and sealed with a metal holder (Neurotar Ltd.) using dental cement.

**Fig. 1:**
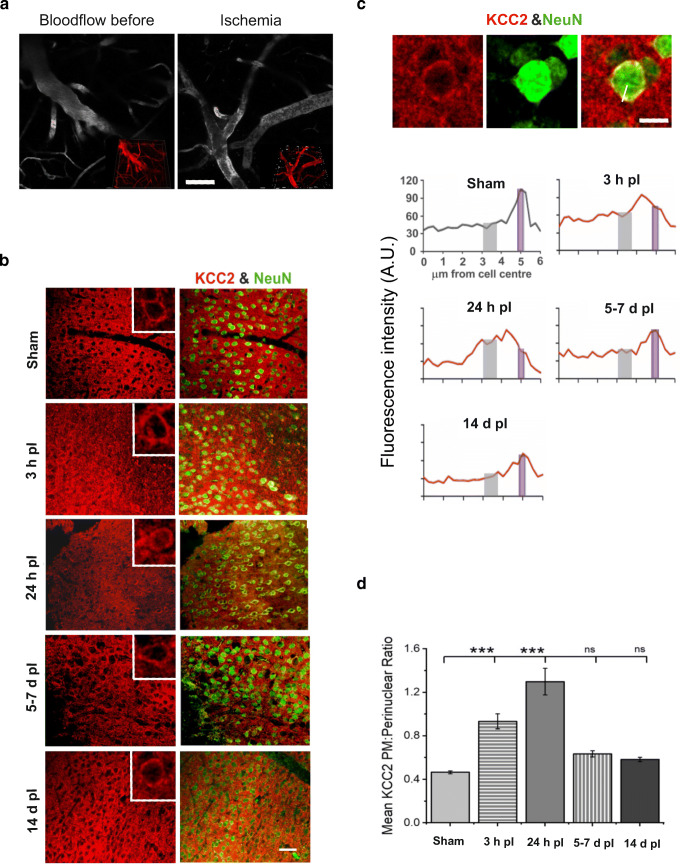
Time course of KCC2 protein changes in PM and overall expression, in neurons within the peri-infarct region following ischemic stroke. **a** 2-photon projection image of cortical blood vessels before and after photothrombotic formation of blood clot. Inlay is the 3D reconstruction of the same vascular region. **b** Representative immunohistochemical stainings in mice brain slices showing KCC2 (red) and NeuN (green) in cells within the stroke peri-infarct zone (cortex), at 3 h (3 h), 24 h (24 h), 5–7 days (5–7 d), and 14 days (14 d) post-ischemia (pI). Large scale bar = 50 μm; insert scale bar = 10 μm. **c** Representative IHC staining of a neuron showing radii from the cell center along which fluorescence intensity was measured (scale bar = 10 μm) (upper panel) and distribution profile of intracellular KCC2 intensity close to the plasma membrane (PM), at different time points post-ischemia (lower panel). **d** Mean ratios of KCC2 staining in perinuclear region (3–3.75 μm from cell center; gray box in **d** to PM KCC2 (estimated at 5–5.25 μm from center; violet box in **d**), obtained from the data in **c**. *n* = 60–90 neurons, from 3 to 4 slices per brain and 3 animals per condition. Data shows mean ± SE. *p* values were calculated by one-way ANOVA. * *p* < 0.05; ** *p* < 0.01; *** *p* < 0.001

Rose Bengal photosensitization was induced in the somatosensory cortex using the photothrombotic method [26] of unilateral focal ischemic stroke with the following modification. After the vein injection of the Rose Bengal solution (10 mg/kg, in PBS), a cortical region, 720 μm in diameter, was illuminated with a green epifluorescent light (535 ± 25 nm) through a × 25/1.05 NA objective (the average power through the objective was 3–10 mW). Photosensitization was performed for up to 5 min until clot formation was confirmed visually in the main vessel. The formation and extent of the clot was followed with a FV1000MPE two-photon microscope (Olympus) using the × 25/1.05 NA (numerical aperture) water-immersion objective as described in Kislin et al. [27] (see Fig. 1a as an example of visualization of clot formation).

### Post-Stroke Treatment

Mice were housed in home cages post-stroke induction and sacrificed at various time points: 3 h (3 h); 24 h (24 h); 5–7 days (5–7 days); and 14 days (14 days), by deep anesthesia and decapitated for slice preparation. For IHC, mice were perfused with PBS and 4% PFA. For the ethanol treatment experiment, animals were given either 3% ethanol-containing or sucrose-containing drinking water, for five consecutive days immediately after ischemic trauma and thereafter sacrificed as above. Average water consumption was not significantly different between groups. Mean consumption during 5 days was (EtOH pI 24.1 ± 3.8 mL and sucrose post-ischemia 26.7 ± 3.8 mL; *p* > 0.5).

### Preparation of Slices for Whole-Cell Patch-Clamp Recordings

Cortical slices were made from sham controls as well as treated and no treated ischemic mice. The animals were anesthetized with pentobarbital, and 400 μm coronal brain slices were cut using a Vibratome 3000 (Vibratome). Slices were bathed in standard physiological solution containing the following (in mM): 124 NaCl, 3 KCl, 2 CaCl_2_, 25 NaHCO_3_, 1.1 NaH_2_PO_4_, 2 MgSO_4_, and 10 D-glucose, equilibrated with 95% O2 and 5% CO2, pH 7.4 at the experimental temperature of 32 °C. The slices were allowed to recover at 36 °C for 1 h before the experiments were started.

### Whole-Cell Patch-Clamp Recordings

The composition of the patch pipette solution was the following (in mM): 18 KCl, 111 K-gluconate, 0.5 CaCl_2_, 2 NaOH, 10 glucose, 10 HEPES, and 2 Mg-ATP, 5 BAPTA, pH 7.3 was adjusted with KOH. The resistance of the patch pipettes was 6.5–7.5 MΩ. The membrane potential values were corrected for a calculated liquid junction potential of 10 mV [28]. KCC2 transport efficiency and local [Cl¯]i were assessed by comparing the somato-dendritic gradients of ΔE_GABA_ induced by local photo-uncaging of GABA [29].

### Local Photolysis of Caged GABA

To assess chloride homeostasis and the dynamics of chloride removal in cortical neurons from post-stroke animals, we used an optical-electrophysiological assay described by Khirug [29]. In brief, neurons were recorded in whole-cell patch-clamp mode, whereupon caged GABA was photolyzed with brief pulses from a UV laser [30, 31] along the dendrite at varying distances from the soma of pyramidal neurons. To expose KCC2 to the Cl- load at soma, we patch-clamped the neurons in whole-cell configuration with 19 mM Cl- in the patch pipette. Values of E_GABA_ were obtained from I–V curves. Under these conditions, experimentally recorded somatic E_GABA_ is very close to the value calculated on the basis of the Nernst equation (− 50 mV). NKCC1 was pharmacologically blocked throughout the experiments by bumetanide (10 μM), a selective blocker of this inward cotransporter at low micromolar concentrations. In addition, action potentials were blocked with TTX (1 μM), and GABA_B_ receptors with CGP 55845 (1 μM). GABA was photolyzed from the carboxy-2-nitrobenzyl (CNB)-caged GABA compound (Invitrogen, Carlsbad, CA) using local uncaging as described previously. Caged GABA (2.5 mM) was dissolved in the physiological solution and delivered at a flow rate of 1 μL/min to the vicinity of the patch-clamped cell using an UltraMicroPump II syringe pump (WPI, Sarasota, FL) and a syringe with an inner tip diameter of 100 μm. For local photolysis of caged GABA, the 375 nm output of a continuous emission diode laser (Excelsior 375, Spectra-Physics) was delivered to the slice through an Olympus LUMPlanFl × 60 water-immersion objective. The UV beam yielded an uncaging spot of ∼ 10 μm diameter [29] that was focused either at the soma or at the dendrite at a certain distance from the soma (50 or 100 μm). In general, less power and shorter flashes were required to induce somatic responses, perhaps mainly because larger membrane surface areas were covered by the uncaging spot at the soma compared with dendrites. At each location, current-voltage relationship was determined by varying the holding potential from − 80 to − 30 mV (10 mV steps). Control experiments showed that changing the voltage-step protocol from hyperpolarizing–depolarizing to depolarizing–hyperpolarizing had no effect on the estimated E_GABA_. Peak current amplitudes were measured and plotted against the holding potential to obtain an estimate of E_GABA_. For each cell, the somatic and dendritic sites were tested in a randomized manner. The gradient (E_GABA_) at the dendrite was defined as the difference of the local value of E_GABA_ and the E_GABA_ at the soma. The gradient (ΔE_GABA_) is calculated as:

$$ \Delta {\mathrm{E}}_{\mathrm{GABA}}=\frac{{\mathrm{E}}_{\mathrm{GABA}}\mathrm{soma}-{\mathrm{E}}_{\mathrm{GABA}}\mathrm{dendrite}}{l} $$

where dendritic *E*_*GABA*_ *soma* is the E_GABA_ at the soma, *E*_*GABA*_ *dentrite* is the E_GABA_ at certain distance *l*, in micrometers from the soma (calculated from the image of the patched neuron). This gives an estimation of the extrusion efficacy of KCC2 in neurons.

### In Vivo Electrophysiological Recordings

Extracellular recordings of spontaneous cortical activity in the somatosensory cortex were performed on urethane (1 g/kg)-anesthetized mice using multisite silicon probes (Neuronexus Technologies, USA) 1 × 16-channel probes with a separation distance of 100 μm between electrodes. The electrodes were placed into the area of the induced stroke or corresponding part of somatosensory cortex of sham animals. The electrode was placed at the depth of the layer 4 that was confirmed by the combination of the following factors: presence of a local field potential (LFP) deflection and spontaneous multiple unit activity (MUA) in the sham animals. Same angle and the depth of electrode were used for post-stroke recordings (Fig. 7b). The analysis was done based on 160 min of recordings per group, two animals in each group. The signals were amplified (× 10,000), filtered (0.1 Hz–10 kHz) using a 128-channel amplifier (NeuroNexus Smartbox, USA) and analyzed post hoc.

Raw data were preprocessed using functions in NeuroExplorer (Nex Technologies). Briefly, raw data were explored to detect MUA, followed by raw data downsampling to 1000 Hz for further analysis of LFP. MUA was detected at a band-passed signal (> 300 and < 6000 Hz) when all negative events exceeding 5 SD were considered as spikes (> 99.9% confidence).

### Immunohistochemistry on Frozen Brain Sections

PFA-fixed brains were cryoprotected in 30% sucrose in PBS, and sectioned into 50 μm slices using the Leica CM 3050S Cryostat. IHC was carried out on free-floating sections. Slices were fixed with 4% PFA-PBS, overnight (ON) at + 4 °C, washed, and dehydrated with 30%, 50%, and 80% methanol (MeOH), respectively, for 30 min in each concentration. Thereafter, they were treated with Dents Fixative (80% MeOH, 20% DMSO) for 1 h and washed twice in TBSTD (TBS + 0.1% Tween + 5% DMSO). Blocking was carried out with 5% Donkey serum and 1% Goat serum (Sigma-Aldrich, D9663 and G9023) in TBSTD ON at + 4 °C. Primary antibodies were applied in the blocking solution for 48 h at + 4 °C. Slices were washed 3 times and secondary antibodies were diluted 1:400 in TBSTD and applied ON. Slices were then washed thrice with TBSTD and mounted onto microscopy glass slides using Prolong Gold mounting medium with DAPI.

### Immunohistochemistry on Fixed Brain Sections

PFA-fixed brains were sectioned into 100-μm slices using the Leica VT1200S. IHC was carried out on free-floating sections. Brain sections were rinsed in PBS and permeabilized with 0.3% Triton-PBS supplemented with 10% normal goat serum (NGS) (Sigma-Aldrich, G9023) for 1 h at RT. Primary antibodies were incubated overnight at 4 °C in 0.3% Triton-PBS supplemented with 3% NGS. Slices were washed 3 times with PBS and secondary antibodies were applied 2 h at RT in 0.3% Triton-PBS supplemented with 3% NGS. Slices were then incubated with Hoechst diluted in PBS (2 μg/mL) for 5 min and washed thrice with PBS. Brain sections were mounted with Fluoro-Gel mounting medium (Electron Microscopy Sciences).

### Antibodies and TG Animals

Rabbit anti-KCC2 pan polyclonal antibodies [32] were produced and purified by Innovagen AB and used at a dilution of 1:1000 for IHC. Primary antibodies Mouse anti-NeuN, clone A60 (Merck Millipore, MAB377, 1:300), mouse anti-GFAP (Merck Millipore, MAB360, 1:500), rabbit anti-Iba1 (Wako, 019-19741, 1:500), and rabbit anti-active® caspase 3 (Promega, G7481, 1:200) were used. The NGFR p75 (MC-192) mouse antibody was from Santa Cruz and was used 1:1000 with the blocking solution from Mouse on Mouse (M.O.M.™) Basic Kit (Vector Laboratories). Secondary antibodies, goat anti-rabbit, or anti-mouse conjugated to Alexa Fluor® 488, 555, 568, or 647 were used at dilutions of 1:500. p75^NTR^ KO mice were obtained from The Jackson Laboratory (Bar Harbor, ME, USA) [33].

### Image Acquisition

Images for data analysis were taken with a Zeiss LSM 710 confocal microscope using the Zen Imaging software or a Leica TCS SP5 X confocal microscope using the Leica Application Suite Advanced Fluorescence (LAS AF) software. With the Zeiss, laser lines used were the argon, diode, and helium-neon, for imaging fluorescent Alexa®488, 568, and DAPI/Hoechst, respectively, with a resolution of 1704 × 1704 (pinhole 1AU) and 4-line average. With the Leica, laser lines used were the diode 405 for Hoechst and the white light laser (WLL) for imaging the different Alexa® fluorescent dyes with a resolution of 1024 × 1024 (pinhole 1AU) and 3-frame average. All the stacks are of about 10-μm thickness. Laser powers, image gain, and other acquisition settings were kept consistent for all images within an experiment.

### Image Quantification and Analysis

For quantification of subcellular distribution of fluorescently labeled KCC2-protein, confocal image stacks were analyzed with ImageJ scientific image analysis software, using a tailor-made macro. Only neurons co-labeled with NeuN were quantified, and those that lay in the peri-infarct area of stroke-brains. Thus, the area of analysis in all lesioned brains was defined as 50–200 μm from the edge of the stroke core. The stroke core was defined as the region of tissue loss (hole), tissue death, or scarring due to the focal ischemic lesion. In sham brains that had no lesion, a corresponding region of the brain section was analyzed. Data was taken from 3 to 4 animals per condition, from 60 to 110 neurons per condition (see individual figure legends for experiment specific *n* numbers). For the NeuN quantification, the same sections analyzed for KCC2 distribution could be used, as they were co-stained with NeuN. For the p75^NTR^ quantification, adjacent brain sections to those used for KCC2 & NeuN analyses were selected for IHC with p75^NTR^ antibody; therefore, the same animals could be analyzed. NeuN cell viability count and p75^NTR^-fluorescence intensity quantifications were carried out using both the ImageJ and Icy image analysis softwares (http://icy.bioimageanalysis.org/). For the fluorescence intensity quantification of GFAP, Iba1, and Caspase 3 in brain sections treated with EtOH or sucrose post-stroke, two circular regions of 75 μm diameter were designed within the core or the penumbra area, fluorescence intensity was measured, and the ratio penumbra/core was calculated. For each slice, this measure was performed twice. Image quantification data were statistically analyzed and all graphs were prepared using the OriginPro 8.6 software or Microsoft Excel 2013. The Shapiro-Wilk test and the Kolmogorov-Smirnov test were used to determine normality of data sets, and statistical significance was calculated using one-way ANOVA, Kruskal-Wallis ANOVA, or the unpaired *t* test. Significance levels and *p* values are shown in individual figure legends.

## Experimental Design and Statistical Analysis

For determining the effect of focal AIS on KCC2 with or without ethanol diet, cell viability (NeuN), p75^NTR^, and electrophysiological recordings, a total of 74 animals were used. All underwent cranial surgery as described above (8 were kept as sham controls); 58 were subjected to laser-induced focal AIS and 24 of these were sacrificed after 3 h, 24 h, 5–7 days, or 2 weeks for further analyses. Three mice from each group of 5 (i.e., from every time point plus sham) were used for in vitro electrophysiology, and the remaining 34 were used for in vivo electrophysiology and IHC. For the ethanol treatment experiment, 18 mice received 3% ethanol in their drinking water for 5 days immediately post-ischemic (pI), and the other 16 received sucrose-containing water for 5 days to control for the additional calories received by the ethanol group.

## Results

### Early Intracellular Post-Ischemia-Induced KCC2 Accumulation Is Followed by Decreased Protein Levels

It is well documented that KCC2 expression and, consequently, GABA-mediated responses are altered in both in vitro and in vivo models of neuropathological conditions such as TLE, TBI, and spinal cord injury [14, 34–38]. Here, we first investigate how and when KCC2 protein expression, and more specifically its subcellular distribution, is affected in the photothrombotic local-ischemia model in mice.

Confocal images of immunostained post-ischemic brain sections, double-labeled with anti-KCC2 and anti-NeuN antibodies, revealed a clearly visible increase in intracellular KCC2 from neurons already at 3 h post-ischemia compared to neurons from sham brains. This transformed into a diffuse and low intensity staining at 24 h post-ischemia that started to return to control distribution at 5–7 days and was almost normal at 14 days (Fig. 1b). In order to estimate the observed putative changes in subcellular distribution, NeuN was used to label neuronal cells and identify their somatic centers. KCC2 localization in relation to the cell center was then evaluated [12]. The mean KCC2 subcellular distribution profiles from control sections showed consistent, low intracellular KCC2-intensity at the cell center which began to rise at approximately 4 μm, peaking between 5 and 5.25 μm from cell center. This peak in KCC2 immunofluorescence intensity profile in control brain sections could be considered to display a low intracellular (perinuclear) expression as compared with a putative close to plasmalemmal (PM) levels. At 3 h post-ischemia, the peak at the putative plasmamembrane was reduced to approximately two-thirds of sham KCC2 intensity, but intracellular values were consistently increased, suggesting that KCC2 protein is rapidly internalized from the plasmamembrane but not yet degraded. It was at the 24-h time point that we saw a marked increase in relative perinuclear KCC2 intensity, accompanied by a further removal of plasmamembrane protein, thus accounting for a possible decrease in functional KCC2 protein. A replenishment of both intracellular and plasmamembrane KCC2 protein levels was noted between 5 and 7 days post-ischemia and showed an almost normal profile at 14 days post-ischemia (Fig. 1c). To interpret the distribution profiles in a more quantitative manner, we calculated the ratio of perinuclear KCC2-intensity (3–3.75 μm from cell center) to plasmamembrane KCC2 (estimated at 5–5.25 μm from center) for all the distribution profiles (Fig. 1d). In healthy neurons, we expected low perinuclear KCC2 and high plasmamembrane KCC2 levels and thus a low perinuclear:plasmamembrane ratio, as calculated from sham brain sections with a mean of 0.47 ± 0.014. This ratio was most significantly increased 3 h post-ischemia to 0.93 ± 0.07 and continued to raise in a statistically significant manner up to 1.30 ± 0.12 at 24 h post-ischemia. Thereafter, the perinuclear:plasmamembrane ratio began to decrease and was significantly lower at 5–7 days post-ischemia compared to 24 h post-ischemia, approaching control ratio values by 14 days post-ischemia. Although the putative plasmamembrane KCC2 protein levels were much lower at 14 days post-ischemia (Fig. 1c; peak fluorescence intensity, 66 ± 2.2 AU) than in sham brains (peak fluorescence, 90 ± 4 AU; *n* = 60–90 neurons, from 3 to 4 slices per brain and 3 animals per condition), the intracellular distribution profiles and perinuclear:plasmamembrane ratios were comparable. These results suggest changes in KCC2-mediated chloride extrusion post-ischemia. Next, we tested if the functionality of KCC2 followed a similar timeline as the putative cellular distribution changes post-ischemia.

### Chloride Extrusion from Neurons and ΔE_GABA_ Are Disrupted Post-Stroke, Recovering by 14 Days

In order to more accurately investigate changes in KCC2-mediated chloride extrusion, we assessed the difference in reversal potential for GABA_A_-mediated responses E_GABA_ between the soma and the distal dendrites upon a slight chloride load at the soma. As previously described [29], this assay unlike the gramicidin-based method where the steady state levels of chloride are estimated mimics a situation where there is a somatic chloride load and discloses the efficacy of chloride extrusion in dendrites.

**Fig. 2:**
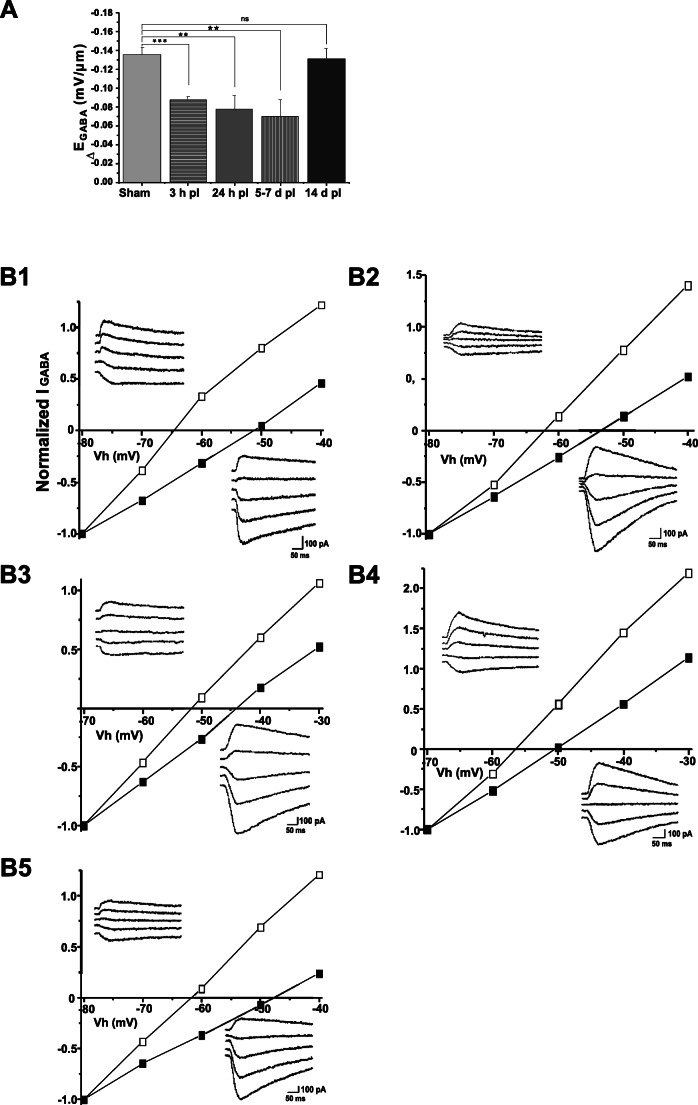
Post-ischemic changes in KCC2 chloride transport efficacy. **a** Graph showing that ΔE_GABA_ values become more positive because of decreased KCC2 Cl¯ extrusion functionality during the first week and then recovers during the second week. KCC2 transport efficiency is expressed here as ΔE_GABA_, that is, the difference between E_GABA_ at dendrites and soma (see the “Material and Methods” section). Data shows mean ± SE. *p* values were calculated by one-way ANOVA. * *p* < 0.05; ** *p* < 0.01; *** *p* < 0.001. Representative current-voltage graphs used to calculate ΔE_GABA_ from sham brains (B1) and in post-ischemia (pI) brains at 3 h pI (B2), 24 h pI (B3), 5–7 days pI (B4), and 14 days pI (B5). Open and closed squares represent currents elicited in the dendrites and in the somas, respectively. *n* = 3–7 neurons were measured per time point, from 15 animals

Normal KCC2 function results in a significantly different E_GABA_ at the dendrites compared to soma, e.g., a lager ΔE_GABA_. As would be expected from the changes in KCC2 subcellular distribution at 3 h post-ischemia, we recorded a significant positive shift (*p* < 0.001; *n* = 5 cells; 3 mice per group) in the ΔE_GABA_ value to − 0.09 ± 0.003 mV/μm compared to − 0.14 ± 0.008 mV/μm observed in sham neurons (*n* = 6 cells; 3 mice per group) (Fig. 2). A positive shift in ΔE_GABA_ implies that the chloride extrusion efficacy is significantly decrease and reflects that GABA_A_ now exerts a more depolarizing action instead of its classical hyperpolarizing action in healthy, mature neurons [15, 29]. The functionality of KCC2 followed a similar pattern to the gradual distribution changes of KCC2 following post-ischemia, except at 5–7 days post-ischemia when we already saw a return of KCC2 protein distribution, but no corresponding increase in KCC2 functionality, and ΔE_GABA_ values (− 0.07 ± 0.017 mV/μm; *n* = 5 cells; 3 mice per group) remaining similar to 24 h post-ischemia (− 0.08 ± 0.015 mV/μm; *n* = 7 cells; 3 mice per group). KCC2 functionality returned to physiological values by 14 days post-ischemia (− 0.13 ± 0.01 mV/μm; *n* = 6 cells; 3 mice per group). These results together with the observed changes in distribution strongly indicate that there are distinct temporal changes in functional KCC2 expression and corresponding GABAergic transmission following post-ischemia.

#### A 3% Ethanol Diet Post-Ischemia Promotes KCC2 Expression and Rescues the Aberrant ΔE_GABA_

**Fig. 3:**
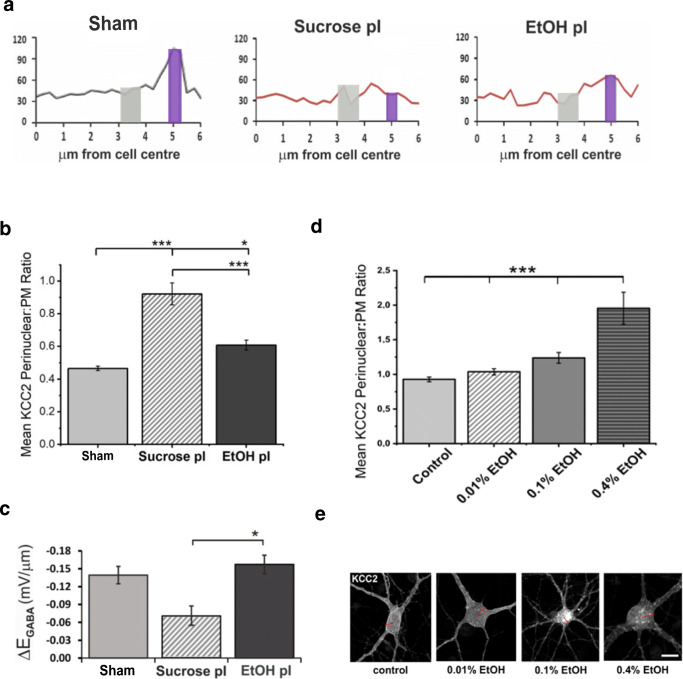
Effect of post-ischemic administration of 3% ethanol in the drinking water for 5 days. **a** Average distribution-profile of KCC2 staining from the cell center to the PM in sham brains, EtOH-treated post-ischemia (EtOH pI), and sucrose-treated post-ischemia (sucrose pI) brains. **b** Average ratios of perinuclear to PM KCC2 from the data gathered in **a**; perinuclear KCC2 (3–3.75 μm from cell center; gray box) to PM KCC2 (estimated at 5–5.25 μm from center; violet box). Both figures above show mean ± SE, from 4 animals per condition and 75–110 neurons per condition. **c** Somato-dendritic gradients of ΔEGABA induced by photo-uncaging of GABA in sham, sucrose pI, and EtOH pI mice brains. *n* = 3, 5, and 3. Data shows mean ± SE. *p* values were calculated by one-way ANOVA. * *p* < 0.05; ** *p* < 0.01; *** *p* < 0.001. **d**–**e** Application of low concentrations of ethanol to cortical cell cultures does not promote KCC2 to PM. **d** Mean ratios of KCC2 staining in perinuclear region (3.25–4.0 μm from cell center) to KCC2 staining at PM, in control cortical cells, and those treated with 0.01%, 0.1%, or 0.4% ethanol (EtOH). Data from 3 separate experiments: *n* = 90–120 neurons per condition. Statistical significance analyzed using the Kruskal-Wallis test; * *p* < 0.05; ** *p* < 0.01; *** *p* < 0.001. **e** Representative ICC images showing KCC2 staining of cortical neurons under each condition and radii along which KCC2 fluorescence intensity was measured (scale bar = 10 μm)

Next, based on Shibasaki et al. [17] finding that low concentrations of ethanol could increase KCC2 levels, we tested if a 3% ethanol diet given post-ischemia to mice could have similar outcome on KCC2. Indeed, compared to a post-ischemia sucrose diet, we noted changes in the subcellular distribution of KCC2 in the brains of mice who received the 3% EtOH in their drinking water for 5 days post-ischemia (Fig. 3a). Although these changes may appear subtle, when expressed in terms of perinuclear:PM ratios (Fig. 3b), we saw that the values quantified in EtOH post-ischemia brains were approaching that of sham brains. Furthermore, the perinuclear:PM ratio in EtOH post-ischemia brains was significantly lowered (*p* = 3 × 10^−6^) compared to sucrose post-ischemia (sham = 0.47 ± 0.014; sucrose pI = 0.92 ± 0.067; EtOH pI = 0.61 ± 0.031; *n* = 6 cells; 3 mice per group).

In terms of KCC2 functionality, the results showed changes that were more robust; the EtOH diet post-ischemia completely rescued the positive shift in ΔE_GABA_ caused by the decrease in Cl¯ extrusion capability due to KCC2 removal (Fig. 3c; sham = − 0.14 ± 0.008 mV/μm; sucrose post-ischemia = − 0.07 ± 0.017 mV/μm; EtOH pI = − 0.15 ± 0.009 mV/μm). Administration of sucrose post-ischemia resulted in a similar ΔE_GABA_ value to that measured at 5–7 days post-ischemia in Fig. 2, confirming that not the additional calories are leading to this recovery.

As it remains unclear from both our and Shibasaki et al. [17] in vivo results, whether the effect of ethanol on neuronal KCC2 is direct or indirect, we assessed if low concentrations of ethanol added to primary neuronal cultures could lead to the same outcome. We found that application of neuronal culture medium containing low EtOH concentrations (0.01%; 0.1% and 0.4% EtOH) to div 7 cortical cells for 3 days promoted small but statistically significant retractions of KCC2 from the PM to the cytosol shown as mean KCC2 perinuclear:PM ratios in Fig. 3d–e. These results, opposing our in vivo findings, suggest that the mechanisms by which ethanol promotes KCC2 expression at the PM in vivo are more complex.

#### Three Percent Ethanol Post-Ischemia Increases Neuron Viability in the Peri-Infarct Region

KCC2 downregulation in both culture systems and certain trauma models has been considered involved in the mechanisms of neuronal cell death via excitotoxicity [14, 39–41].

**Fig. 4:**
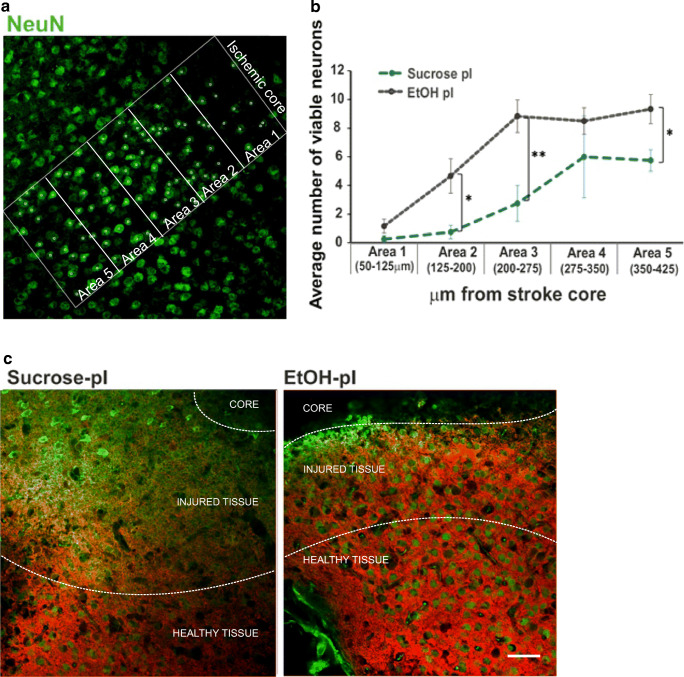
Effect of post-stroke EtOH administration on neuron viability in the ischemic penumbra. **a** Representative IHC showing NeuN staining in an EtOH pI brain section. A number of NeuN-positive cells (green circles) in each area (white rectangle) were counted, with area 1 being positioned closest to the stroke core and area 5 the furthest. The same method of quantification was repeated for sucrose pI sections. **b** Quantification of the average number of viable neurons (NeuN-positive cells) in areas 1–5, situated at various distances from the stroke core. Mean ± SE, from 3 to 4 animals per condition. *p* values were calculated by *t* test. * *p* < 0.05; ** *p* < 0.01; *** *p* < 0.001. **c** Representative IHC images (green = NeuN, red = KCC2). Scale bar = 50 μm

Since the ethanol diet rescued the diminished GABA_A_-mediated inhibition post-ischemia, we examined if it affected neuronal survival in the IS peri-infarct zone. We assessed neuron viability by the NeuN immunostaining particularly close to the stroke core. NeuN-stained cells were counted inside five equally sized areas arranged progressively further from the stroke core (Fig. 4a). Ethanol-treated, post-ischemic slices (EtOH post-ischemia) retained a greater number of their neurons than sucrose post-ischemia slices (sucrose post-ischemia), in all but the 1st area, closest to the stroke lesion, which comprised mostly scar tissue (Fig. 4b). The most pronounced effect of EtOH post-ischemia treatment was observed in areas 2 and 3 (125–275 μm from the stroke core) showing an approximate four-time increase in cell number compared to sucrose post-ischemia. Presumably, the most substantial neuronal loss occurred in this region (number of NeuN-viable cells; sucrose pI, area 1 = 0.25 ± 0.25, area 2 = 0.75 ± 0.48, area 3 = 2.75 ± 1.25, area 4 = 6 ± 2.86, area 5 = 5.75 ± 0.75; EtOH pI, area 1 = 1.17 ± 0.48, area 2 = 4.67 ± 1.2, area 3 = 8.83 ± 1.14, area 4 = 8.50 ± 0.92, area 5 = 9.33 ± 1.02; *n* = 3 and 4 animals, respectively) if compared to the number of NeuN-positive cells in sham cortex (20.3 ± 3.14; *n* = 4 animals; see Fig. 1b). Figure 4c shows characteristic IHC stainings from EtOH post-ischemia and sucrose post-ischemia brain sections, from which it is apparent that the area of disrupted NeuN and KCC2 staining or injured tissue surrounding the stroke core is reduced in EtOH post-ischemia slices.

**Fig. 5:**
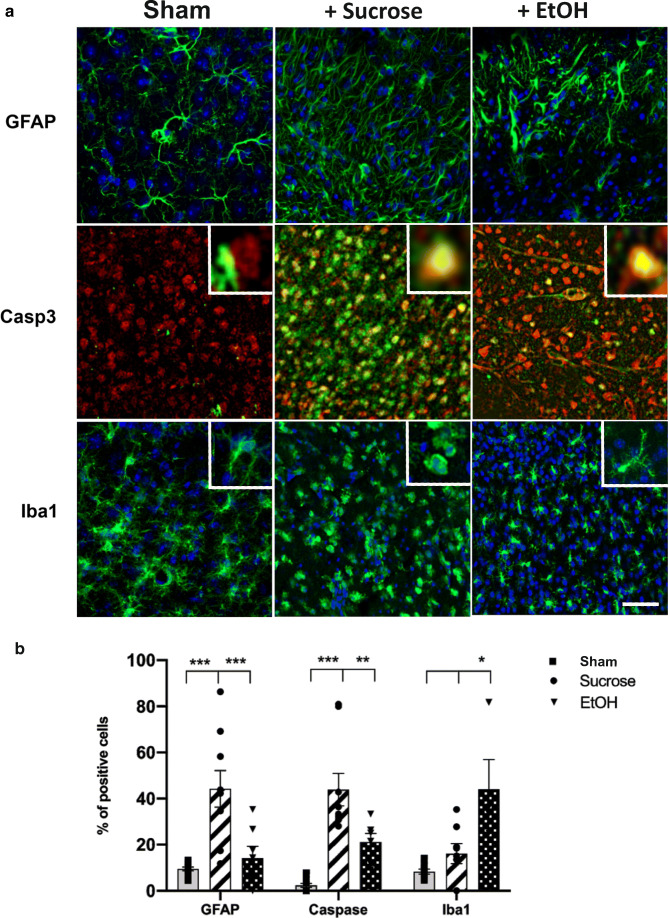
Effect of post-ischemia (pI) EtOH administration on markers for apoptosis and inflammation. **a** Representative immunohistochemistry showing GFAP (top), Caspase 3 (middle), Iba1 (bottom) staining (green), or NeuN staining (red) in brain sections from sham (left), sucrose pI (middle), or EtOH pI (right). Nuclei were labeled with Hoechst (blue). Each staining clearly delineated the region of the stroke. After EtOH treatment, we observed a total absence of GFAP positive cells within the stroke core, less Casp3 positive cells, and less ameboid microglia. In the Iba1 staining, the inset images show an ameboid (left) and a ramified microglia (right) (scale bar = 100 μm). **b** For GFAP and Caspase 3 staining, number of positive cells was counted in circular regions of 75 μm diameter and normalized with the total number of cells. For the Iba1 staining, only the ramified positive cells were counted. Measurements were performed in 2 to 5 slices from 4 to 3 animals treated post-ischemia with sucrose or EtOH, respectively. Data are expressed as means ± sem. *p* values were calculated by unpaired *t* test. * *p* < 0.05

Consistent with the increased neuronal survival induced by EtOH, examination of IHC against the apoptotic marker-cleaved caspase-3 shows reduced number of positive cells within the core after EtOH treatment compare to sucrose (EtOH post-ischemia, 21.1 ± 3.8; sucrose post-ischemia, 43.9 ± 7.1; Fig. 5). Ischemic stroke is characteristically followed by changes in reactive microglia as well as astrocytes. Sections from ethanol-treated animals showed significant changes in the distribution of both Iba1 and GFAP. GFAP staining showed a strong decrease in the number of cells in the core zone after EtOH treatment (sucrose post-ischemia, 44.3 ± 8.0; EtOH post-ischemia, 14.2 ± 4.4, Fig. 5). We also analyzed the microglia morphological changes with the Iba1 staining and showed an increase in the number of ramified positive cells which correspond to the resting microglia (sucrose post-ischemia, 16.1 ± 4.3; EtOH post-ischemia, 44.1 ± 12.8, Fig. 5). These results indicate that ethanol administration has significant impact on the trauma-induced distribution and activation of both microglia and astrocytes. It is plausible that this effect may contribute to the mechanism leading to the amelioration of qualitative changes in GABAergic transmission and increased neuronal survival.

### An Increase in p75^NTR^ in the Stroke Penumbra at Day 5 Post-Stroke Is Reduced by the 3% Ethanol Diet

A body of data, including our own previous work, demonstrates a functional correlation between an upregulation of the pan-neurotrophin receptor p75^NTR^ and neuronal cell apoptosis both post-trauma [42, 43] and in neurodegenerative disease models [44]. The mechanism for trauma-induced upregulation of p75^NTR^ is dependent on downregulation of KCC2 and consequent GABA_A_-mediated depolarization [42].

**Fig. 6:**
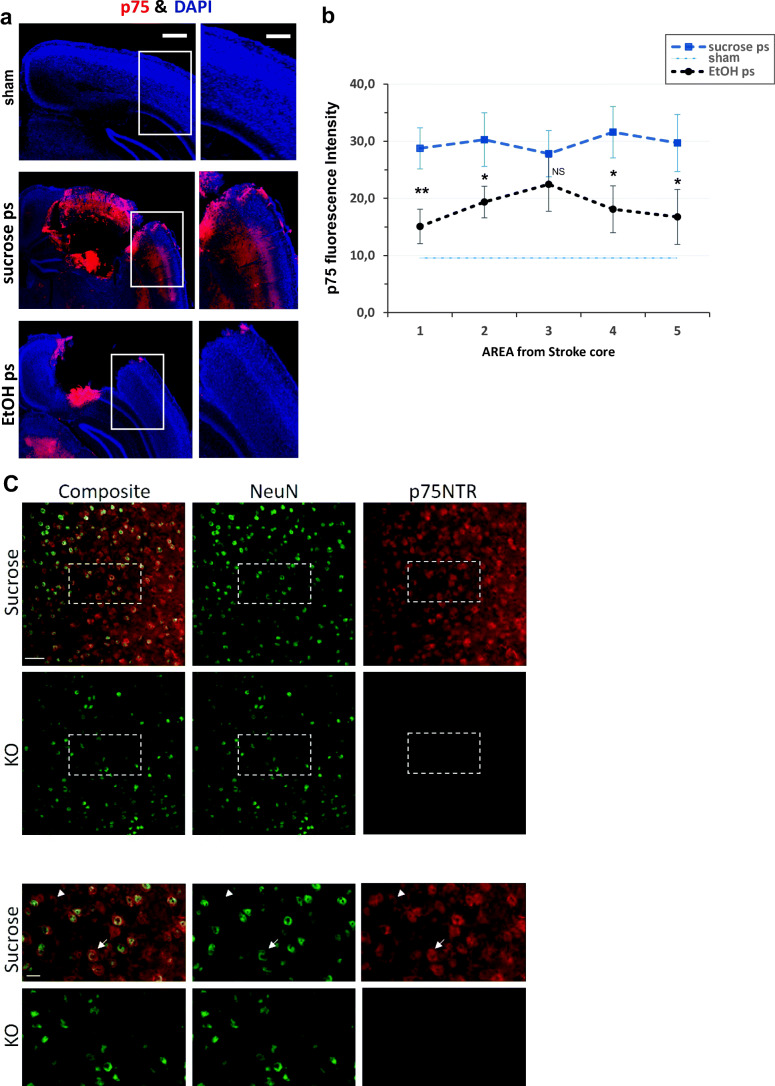
Post-ischemia induced upregulation of p75^NTR^ was rescued by EtOH diet but not by sucrose. **a** Representative immunohistochemistry of brain sections stained against p75^NTR^ (red) and DAPI (blue) from either sham, sucrose diet, or EtOH diet animals 5 days after ischemia (scale bar = 500 μm, insert = 250 μm). **b** Quantification of p75 ^NTR^ fluorescence intensities in areas 1–5 in both sucrose pI (blue-dashed line) and EtOH pI (black-dotted line) mice brain sections (area 1 being closest to stroke core and area 5, the furthest as used for NeuN cell viability quantification in Fig. 4). Mean ± SE, *n* = 7 per condition. *p* values were calculated by *t* test. NS, not significant; * *p* < 0.05; ** *p* < 0.01. **c** Low magnification images of p75^NTR^ and NeuN immuno-like reactivity in sections from WT PI animals treated with sucrose (uppermost panel) and sham p75^NTR^ KO animals (lower panel). Lower most panels show high magnification images from regions demarked in corresponding upper panels in **c** (scale bar; upper panel = 100 μm, lower panel = 25 μm)

Here, we analyzed the p75^NTR^ expression levels in the stroke penumbral region 5 days post-ischemic trauma by immunohistochemistry and compared the group of mice that were given the EtOH diet to those on the sucrose diet or (Fig. 6a). We observed a marked increase in p75^NTR^ expression at day 5 in sucrose post-ischemia brains compared to EtOH post-ischemia and sham cortex (9.75 ± 2.5) brains, which was most pronounced in area 1 (50–125 mm from core) in which the fluorescence intensity was almost halved in the EtOH post-ischemia mice brains compared to sucrose post-ischemia (Fig. 6b). p75^NTR^ fluorescence intensities: sucrose post-ischemia (areas 1–5, respectively, 28.8 ± 3.6; 30.3 ± 4.7; 27.8 ± 4.0; 31.6 ± 4.5; 29.7 ± 5). EtOH post-ischemia (15.1 ± 3.0; 19.4 ± 2.8; 22.5 ± 4.7; 18.1 ± 4.1; 16.8 ± 4.8; *n* = 7, *p* < 0.05; *n* = 4 animals per condition). High-magnification images showed that the majority of cells with higher p75^NTR^ expression were also positive for NeuN 86.92% (NeuN *n* = 306 and NeuN plus P75 *n* = 266). The specificity of the staining was tested in slices from p75^NTR^ KO (NeuN *n* = 224 and NeuN plus P75 *n* = 0) (Fig. 6c). The reduced p75^NTR^ expression profile observed in EtOH-treated animals agrees with the reduced neuronal apoptosis induced by EtOH. The neuronal like morphology of p75^NTR^ positive cells correlates with the inhibition of post-ischemia-induced neurospecific KCC2 decrease and functional recovery of GABA_A_-mediated responses.

### In Vivo Electrophysiological Recordings Reveal EtOH-Induced Functional Recovery of Cortical Layer IV

**Fig. 7:**
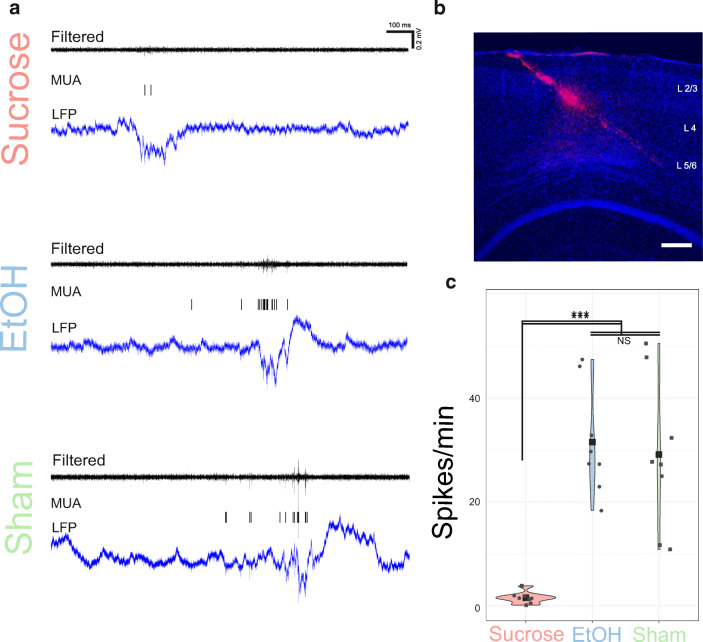
In vivo intracortical recording show functional recovery after EtOH administration. **a** Local field (LFP) recordings (lower trace) and corresponding multiunit activity (MUA) in sucrose, EtOH-treated, or sham animals. **b** Position of recording probe in the somatosensory cortex (250 μm). **c** Quantification of frequency of MUA. Mean ± SE, from 2 animals per condition. *p* values were calculated by *t* test. NS not significant; *** *p* < 0.001)

The layer IV of the somatosensory cortex is a major target of the thalamic input and plays an important role at early sensory information processing [45]. During physiological conditions and under urethane anesthesia, the frequency of multiunit activity (MUA) is typically highest during negative potential shifts also referred as cortical upstates [46]. In vivo spontaneous MUA frequency (Fig. 7a) recorded from the L4 displayed a typical pattern with increased frequency during negative potential shifts (Fig. 7b), this was significantly lower in the sucrose-treated animals (1.58 ± 0.4; *n* = 2; *p* < 0.001) as compared to sham (29.12 ± 5.1; *n* = 2; *p* < 0.001). This is most likely the consequence of a massive post-stroke neuronal damage as indicated by immunohistochemistry of the cortical layers. Interestingly, in EtOH-treated animals, the MUA frequency (31.48 ± 3.7; *n* = 2; *p* < 0.001) was not different from the sham group (*p* = 0.7) (Fig. 7c). We can conclude that EtOH treatment for 5 days ameliorates functional deterioration of an important functional circuit of the somatosensory cortex.

## Discussion

For acute ischemic stroke (AIS), IV recombinant tissue plasminogen activator (rtPA) therapy is currently the only FDA-approved treatment available to patients. However, the narrow administrative time window and unsuitability limit its availability for the vast majority of AIS patients [47].

Motivated by the recent explosion of interest in the potassium-chloride cotransporter, KCC2, and its role in different brain diseases including trauma, here, we investigate the changes in expression as well as functional consequences in a focal AIS mouse model. Primarily, we discovered a new avenue of potential therapy based on the rescue of the altered GABAergic transmission and neuronal cell loss.

It has been reported in numerous studies that after certain injuries, the [Cl¯]_**i**_ raises and compromises the GABA_A_-mediated inhibitory responses in neurons (reviewed by [48]. Similarly, a few studies have shown changes in KCC2 expression in different stroke models [19, 40, 49]. The results presented here, however, show for the first time that it occurs also after AIS in the stroke penumbra of cortical structures.

The retraction of plasmamembrane KCC2 into the cytosol of neurons is already observed at 3 h post-ischemia, rendering these neurons at risk for excitotoxicity and death due to diminished or reversed GABAergic responses. Between 3 and 24 h post-ischemia, the decrease in overall KCC2 levels is likely a result of protein degradation in the neurons within the peri-infarct region [48]. The fate of the neuron may therefore depend on whether KCC2 protein is degraded during this time window of 3–24 h post-ischemia [38, 41]. By 5–7 days post-ischemia, the neurons within the peri-infarct area that have managed to survive seem to recover as the KCC2 protein is again translated and trafficked to the PM.

Normal chloride extrusion capacity in dendrites only return to physiological levels at 14 days post-ischemia as reflected by the changes in ΔE_GABA_ values, suggesting that the plasmamembrane re-expression of KCC2 protein occurs at an earlier time point than the functional recovery of neuronal Cl¯ extrusion. This implies that additional factors, which participate in determining the fate of the neuron, may be required to take place after the 5–7 days post-ischemia time point, before plasmamembrane KCC2 is functionally activated. It should be noted that the method used to assess chloride extrusion primarily monitors the extrusion efficacy at the dendrites due to the configuration of the recording. Thus, it is also possible that temporal recovery of KCC2 differs between soma and dendrites. A more detailed analysis using, e.g., dendritic tracing would be required to establish if there would be a difference.

The most intriguing finding in this study demonstrated that ethanol administered for 5 days post-ischemia promotes upregulation of KCC2 protein at the plasmamembrane and enhances Cl¯ extruding functionality. The outcome of the ethanol treatment was also interpreted in terms of neuronal viability, showing a clear increase in cell count in the region surrounding the ischemic core. Further investigation of this possible treatment avenue is certainly justified, especially as ethanol would provide an extremely inexpensive and easily available option for all parts of the world.

Several recent studies show that in the adult, there are indications that ethanol could have positive effects particularly for the traumatized brain [3–10, 50]. Some of the mechanisms proposed to mediate this positive effect of low concentrations of ethanol are to suppress apoptosis and inflammation. Interestingly, the effect of ethanol at different concentration in the in vitro preparation of primary cortical cultures induced a decrease in the plasmamembrane-like KCC2 expression.

Subsequently, we chose to investigate possible routes through which EtOH, administered post-stroke, exerts the observed effects. We have previously shown, in an acute injury model [42], that upregulation of p75^NTR^ can occur as a consequence of depolarizing GABAergic currents post-trauma. In the current work, we were able to reverse the depolarizing GABA-mediated transmission with the post-ischemia EtOH treatment. It is plausible that ethanol may assert its effect by inducing re-expression of KCC2 at the plasmamembrane and reversing GABA-mediated responses to hyperpolarizing, thus reducing the excitability-induced upregulation of p75^NTR^. Additionally, it was recently shown that blocking the proBDNF- p75^NTR^ pathway post-SE, using an antibody against p75^NTR^ could restore KCC2 cellular distribution [22]. Therefore, another possibility is that ethanol may assert its effect on p75^NTR^ although this is unlikely as the effect of ethanol on P75^NTR^ expression appears to depend on the developmental stage of neurons [51]. By whichever means, this reduction of p75^NTR^ expression during the acute stages (up to 5 days post-ischemia) seems important for reducing the number of dead neurons in the penumbra. It may serve to enhance the protective element necessary after an injury. Only after this phase, renewal and regenerative elements such as axonal sprouting can begin which require very different molecular triggers and may even necessitate additional p75^NTR^ levels as found during development [52].

It is well known that ethanol modulates currents at GABA_A_ and glycine receptors [53], but less well known are its trophic effects which can promote dendritic growth in certain cases [54]. More relevantly, an interesting study looking at the sensitization of mice to morphine found that an addition of 3% ethanol to the diet increased KCC2 expression in the mice brain [17]. Our findings from primary cortical cells with low concentrations of ethanol suggest a different mechanism of ethanol on KCC2 in vivo that leads to an increase in functional plasmamembrane KCC2 after AIS. It may be possible that ethanol exerts its effects via cell types less prominent in primary cell cultures as for example microglia and astrocytes. Interestingly, unlike the effect on the developing brain where ethanol can induce transient activation of microglia resulting in phagocytosis of degenerating neurons, and a prolonged increase in glial fibrillary acidic protein-positive astrocytes activation as described by Saito el at [55], the results presented in this study in adult brain following trauma are the opposite. Remarkably, a very recent report investigating the neuroprotective effect of acute ethanol intoxication in TBI discovered that it downregulated some immediate early genes including those that transcribe BDNF [50]. BDNF, acting through TrkB receptors in mature neurons, is known to cause retraction of KCC2 from the plasmamembrane and hence reversal in polarity of currents via GABA [34, 38, 56].

The positive effects of low concentration of ethanol was also observed on in vivo neuronal firing as demonstrated by the significant amelioration of trauma-induced decrease in unit activity in the affected somatosensory cortex. This is consistent with the reduced cell death promoted by EtOH.

To summarize, we have found a novel, therapeutically relevant positive effect of low levels of ethanol on post-ischemic neuronal survival that may involve the upregulation of KCC2 as well as qualitative changes in GABAergic transmission and downregulation of p75^NTR^.
